# Extracellular vesicles released during hypoxia transport heparanase and enhance macrophage migration, endothelial tube formation and cancer cell stemness

**DOI:** 10.1002/pgr2.1

**Published:** 2023-03-28

**Authors:** Kaushlendra Tripathi, Shyam K. Bandari, Ralph D. Sanderson

**Affiliations:** ^1^ Department of Pathology University of Alabama at Birmingham Birmingham AL USA; ^2^ Present address: Building 29B, Room 5NN Suite 22, Lab 5NN11, Molecular Pathology Section Lab of Immunogenetics, NIAID, NIH 9000 Rockville Pike Bethesda Maryland 20892 USA; ^3^ Present address: Exelixis 1851 Harbor Bay Parkway Alameda California 94502 USA

**Keywords:** exosome, heparanase, hypoxia, macrophages, myeloma, stem cells, tumor

## Abstract

Heparanase is upregulated during the progression of most cancers and via its enzyme activity promotes extracellular matrix degradation, angiogenesis and cell migration. Heparanase expression is often associated with enhanced tumor aggressiveness and chemoresistance. We previously demonstrated that increased heparanase (HPSE) expression in tumor cells enhances secretion and alters the composition of tumor‐released exosomes. In the present study, we discovered that extracellular vesicles (EVs) secreted by human multiple myeloma cells growing in hypoxic conditions exhibited elevated levels of HPSE cargo compared to EVs from cells growing in normoxic conditions. When macrophages (RAW 264.7 monocyte/macrophage‐like cells) were exposed to EVs released by tumor cells growing in either hypoxic or normoxic conditions, macrophage migration and invasion was elevated by EVs from hypoxic conditions. The elevated invasion of macrophages was blocked by a monoclonal antibody that inhibits HPSE enzyme activity. Moreover, the HPSE‐bearing EVs from hypoxic cells greatly enhanced endothelial cell tube formation consistent with the known role of HPSE in promoting angiogenesis. EVs from hypoxic tumor cells when compared with EVs from normoxic cells also enhanced cancer stemness properties of both CAG and RPMI 8226 human myeloma cells. Together these data indicate that under hypoxic conditions, tumor cells secrete EVs having an elevated level of HPSE as cargo. These EVs can act on both tumor and nontumor cells, enhancing tumor progression and tumor cell stemness that likely supports chemoresistance and relapse of tumor.

**Abbreviations**
EVs: extracellular vesicles
FBS: fetal bovine serum
HIF: hypoxia‐inducible factor
HPSE: heparanase
VEGF: vascular endothelial growth factor

## INTRODUCTION

Hypoxia in cancer results from excessive tumor cell proliferation leading to increased oxygen consumption and the absence of sufficient blood supply to support tumor growth.^1^ Central to the effects of hypoxia in cancer is the activation of the hypoxia‐inducible factor (HIF) signaling pathway.^2^ HIF‐1α subunits translocate to the nucleus and form a heteromeric complex with HIF‐1β that then interact with hypoxia‐responsive elements. The result is upregulation of an array of genes that promote tumor growth, angiogenesis, migration, and invasion.^3^, ^4^, ^5^ Moreover, the establishment of hypoxic zones within the tumor microenvironment support cancer stem cells thereby enhancing tumor chemoresistance and contributing to poor patient survival.^6^ This support of cancer stem cells in part is due to the activity of HIF1α that is released in hypoxic conditions.^7^ In addition, hypoxia enhances cell growth, self‐renewal, and tumorigenesis, consistent with the characteristics of cancer stem cells.^8^

Recently there has been increasing interest in the role of EVs and their relationship to hypoxia. Multiple studies have indicated that EV release increases when cells are growing in hypoxic conditions.^9^ Of particular interest are studies in an array of cancer types demonstrating that EVs from hypoxic cells have the capacity to promote tumor cell migration, invasion, proliferation, and stemness.^9^, ^10^, ^11^ Exosomes are EVs that range in size from ~50 to 200 nm and have been widely studied in cancer. They comprise a broad heterogeneous population of vesicles that even from the same cell type can vary in cargo and function thus representing layers of complex functional potential.

Our laboratory has studied the role of the heparanase (HPSE) and exosomes in regulating the behavior of myeloma tumor cells. Heparanase is an enzyme that degrades heparan sulfate and serves as a master regulator of the aggressive tumor phenotype and crosstalk within the tumor microenvironment.^12^, ^13^ We discovered an important role for HPSE in regulating exosome biogenesis, composition and function.^14^ We also found that exosomes released by myeloma cells expressing a high level of HPSE could deliver exosomal HPSE to recipient cells thereby promoting tumor cell spreading, endothelial cell invasion and enhanced stem cell formation.^14^, ^15^ These functional capacities of exosomes can be blocked by inhibiting HPSE enzyme activity. Moreover, following treatment of myeloma cells with drugs used routinely to treat myeloma patients (e.g., bortezomib), exosome secretion was enhanced and the exosomes had an elevated level of HPSE cargo. When incubated with myeloma cells not exposed to drug, the HPSE cargo was delivered to the cells and stimulated the extracellular signal‐regulated kinase signaling pathway, a pathway known to enhance chemoresistance.^16^, ^17^

In the present study, we isolated extracellular vesicle (EVs) secreted by myeloma cells growing in either normoxic or hypoxic conditions and examined their potential to impact cells in the tumor microenvironment, including other tumor cells. We discovered that EVs secreted by hypoxic cells carried a high level of HPSE cargo. These EVs promoted macrophage migration and invasion, endothelial tube formation and induction of a stem cell‐like phenotype in myeloma cells. In contrast, EVs secreted by cells growing in normoxic conditions had a much less dramatic functional impact. Together, these findings indicate that under hypoxic conditions, myeloma cells secrete EVs carrying elevated levels of HPSE that can enhance aggressive tumor behavior and promote cancer stemness that likely enhances chemoresistance.

## MATERIALS AND METHODS

### Cell lines and reagents

CAG human myeloma cells were isolated as described.^18^ Roswell Park Memorial Institute (RPMI) 8226 myeloma cells and RAW 264.7 (RAW) monocyte/macrophage‐like cells were obtained from ATCC. RAW cells originated from an Abelson leukemia virus‐transformed cell line derived from BALB/c mice.^19^ All cell lines were expanded and frozen in multiple vials upon receipt. All experiments were carried out within 6 weeks of thawing cells and not passaged more than five times. Cells were routinely tested for mycoplasma. The generation of CAG cells expressing a high level of heparanase (CAG‐HPSE) has been described.^20^ The level of HPSE activity in CAG‐HPSE cells is comparable to levels detected in bone marrow of many myeloma patients and thus represent a physiologically relevant model for studying HPSE function in myeloma.

### Cell culture

Myeloma cell lines were cultured at 37°C in a 5% CO_2_ humidified environment in RPMI1640 medium supplemented with 10% fetal bovine serum (FBS) and 100 U/ml penicillin G and 100 µg/ml streptomycin sulfate. For hypoxia experiments, cells were grown in a hypoxia chamber (Coy Laboratory Products) at 1% O_2_ at 37°C in a 5% CO_2_ humidified environment.

### EV isolation

EVs were isolated from the medium conditioned by CAG‐HPSE cells as previously described.^14^ Briefly, cells were cultured for 48 h, washed twice and placed in RPMI 1640 medium lacking FBS and grown in either normoxic (21% O_2_) or hypoxic (1% O_2_) conditions for 24 h. Conditioned media was harvested and EVs isolated using serial centrifugation at low speed, passed through a 0.2 µ filter followed by ultracentrifugation (L‐80 Ultracentrifuge, Beckman Coulter) at 40,000 rpm (196,768*g*) using type 70.1 Ti fixed angel rotor (Beckman Coulter). The pellet was resuspended in phosphate buffered saline (PBS) and centrifuged again at 40,000 rpm and the pellet resuspended in PBS. Isolated EVs were subjected to nanoparticle tracking analysis using the NanoSight LM10 system (NanoSight Ltd) equipped with a blue laser (405 nm). Particle movement via Brownian motion of the laser‐illuminated nanoparticles was captured for 60 s. The recorded video was analyzed using NanoSight particle tracking software to calculate nanoparticle concentration and size. This analysis of isolated particles revealed a profile consistent with the characteristics of exosomes.^16^ However, in the present study these particles were not further characterized and are thus referred to as EVs rather than exosomes.

### Western blot

For western blot analysis, EVs from each sample were isolated, analyzed, and quantified by NanoSight analysis and heated to 100°C for 15 min in sodium dodecyl sulfate‐polyacrylamide gel electrophoresis (SDS‐PAGE) buffer. The equivalent of 2 × 10^6^ EVs were loaded into each well of an SDS‐PAGE gel. Gels were electroblotted onto nitrocellulose membrane and blocked 1 h in Tris‐buffered saline containing 5% milk powder and 0.1% Tween‐20. Membranes were incubated with anti‐HPSE polyclonal antibody^21^ for 2 h, followed by peroxidase‐conjugated secondary antibody in Tris‐buffered saline containing 0.1% Tween‐20 for 1 h. Bound antibody was visualized using a chemiluminescence kit from Milipore.

### Scratch assay

The effect of EVs secreted by myeloma cells growing in normoxic or hypoxic conditions on RAW cell migration was examined. RAW cells were grown to confluence in six‐well plates and pipette tips were used to scratch cells from the surface of the plate. Plates were washed twice with media to remove detached cells. Photomicrographs of initial wounds were taken using Canon Power Shot A640 digital camera (at ×100 magnification). Serum‐free medium (2 mL) was added to each well along with 4 × 10^6^ EVs secreted by CAG‐HPSE cells growing in either normoxic or hypoxic conditions. After 6 h, complete medium was added to each well and cells placed back in the incubator for 16 h. Photomicrographs were taken and ImageJ software was used to determine the percent area covered by cells within each scratch.

### Invasion assay

Assays utilized matrigel‐coated trans‐well chambers from BD Biosciences. In this assay, bottom chambers were filled with RPMI medium with 10% FBS depleted of EVs (System Biosciences). RAW cells (1 × 10^5^ in each well of the 12‐well plate) were introduced to the upper chamber and the following day 1 × 10^6^ EVs per well were added to the upper chamber. In some experiments, the anti‐HPSE monoclonal antibody H1023 (15 µg/mL) was incubated with EVs for 4 h before their incubation with the RAW cells. After 24 h, cells on the top surface of the membranes (noninvasive cells) were scraped with cotton swabs and cells on the bottom surface of the membranes (invasive cells) were fixed with cold methanol, stained with hematoxylin/eosin and mounted. Images were taken using Cannon Power Shot A640 camera on Zeiss inverted microscope, and invasive cells were counted at ×100.

### Endothelial tube formation assay

Human umbilical vein endothelial cells were treated with EVs harvested from media conditioned by CAG‐HPSE myeloma cells growing in normoxic or hypoxic conditions and an in vitro tube formation assay was performed in 96‐well plates using an angiogenesis kit (Millipore) as per manufacturer protocol. Twelve hours after plating cells, tube formation was examined by microscopy and tube length and number of branch points were quantified using ImageJ software.

### Sphere formation assay

Wild‐type CAG cells that express a low level of HPSE growing in normal cell culture conditions were harvested, counted and seeded at density of 1 × 10^4^ cells/well, then subjected to sphere cluster formation assay as per the earlier report.^15^, ^22^ In brief, the cells were carefully dispersed as single cells and cultured for 10–12 days in stem cell specific, serum free medium (2 mL) in ultra‐low attachment six‐well plates (Costar). This defined medium was DMEM/F‐12 (1:1 ratio) supplemented with 1% penicillin–streptomycin, B27 and N2 supplements and growth factors (recombinant human epidermal growth factor and fibroblast growth factor). This medium supports the growth of stem cells, and with time, cells proliferate to form floating clonal spheres. The medium was replaced every 72 h. After seeding, cells were observed daily to ensure that spheres formed because of cell multiplication and not due to adherence and clustering of adjacent cells. The stem spheres with greater than 50 cells were scored as large (true stem cell spheres), while spheres less than 50 but greater than 15 cells were scored as small spheres.

### Statistical analysis

Results were analyzed with analysis of variance software using a two‐tailed *t*‐test. A *p* ≤ 0.05 was considered significant.

## RESULTS

### EVs secreted by hypoxic cells enhance macrophage cell migration and invasion

**Figure 1:**
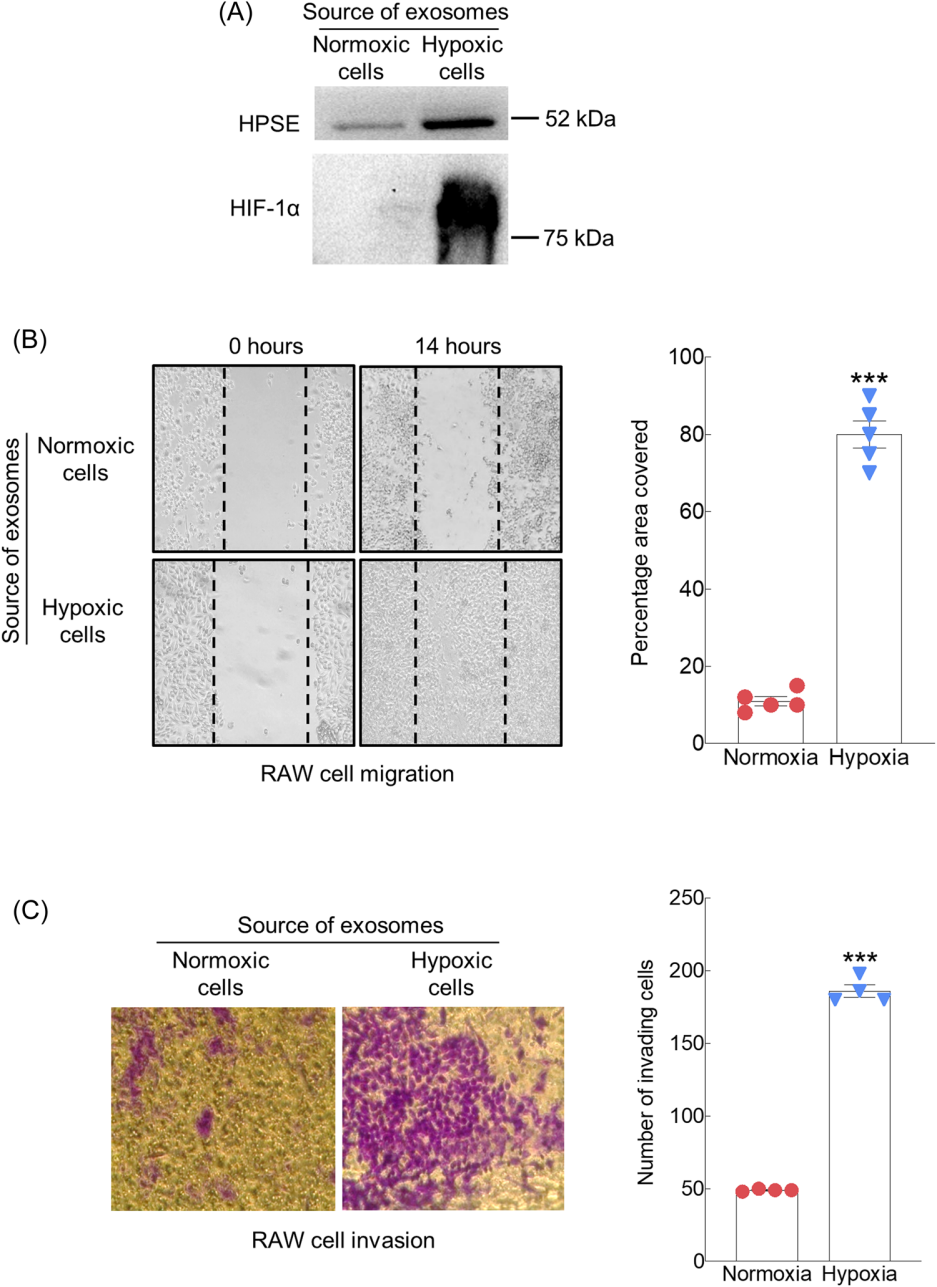
Hypoxic environment promotes secretion of EVs that enhance migration and invasion of RAW macrophages. (A) CAG‐HPSE myeloma cells were grown in normoxic or hypoxic conditions for 48 h. The secreted EVs were isolated, analyzed and quantified by NTA, extracted, subjected to SDS‐PAGE, and western blots probed with antibody to human heparanase^21^ or HIF‐1α. The equivalent of 2 × 10^6^ EVs were loaded into each well. (B) EVs secreted by CAG‐HPSE myeloma cells growing in either normoxic or hypoxic conditions were incubated with RAW cells and cell migration was quantified in scratch assays. An equal number of EVs were added to each chamber. Shown are representative photomicrographs taken at 0 h and 14 h following the introduction of EVs (original magnification, ×100). Dotted lines denote boundaries of the scratch at 0 h. Percent of the area covered by cells within the scratch region was determined by ImageJ software. Three separate experiments were performed, and percent of the area covered is shown from triplicate wells of a single representative experiment (right panel). ***p ≤ 0.001. (C) EVs secreted by CAG‐HPSE myeloma cells grown in either normoxic or hypoxic conditions were isolated and added in equal number to RAW cells that were plated on matrigel in a transwell invasion assay. Cells migrating through the filter were stained blue, photographed and counted. Representative photomicrographs show cells that had invaded 16 h after introduction of EVs (Original magnification, ×100). Three separate experiments were performed and numbers of invading cells shown are from triplicate wells of a single representative experiment (right panel). ***P ≤ 0.001. EV, extracellular vesicle; HIF, hypoxia‐inducible factor; NTA, nanoparticle tracking analysis.

Upregulation of HPSE expression has been associated with hypoxic conditions in numerous biological systems.^23^, ^24^, ^25^, ^26^ In addition, we and others have demonstrated that upregulation of HPSE expression promotes exosome biogenesis and secretion of exosomes carrying detectable amounts of HPSE as cargo.^14^, ^27^ Based on these previous findings, we speculated that exposing myeloma tumor cells to a hypoxic environment would result in the release of EVs having an elevated level of HPSE as cargo. To test this, human myeloma CAG cells that express heparanase (CAG‐HPSE) were grown under normal cell culture conditions (21% O_2_, normoxia) or under low oxygen conditions (1% O_2_, hypoxia) for 48 h. In normoxia, the EVs released had a relatively low level of HPSE, present as the ~50 kDa active form of the enzyme (Figure 1A). In contrast, EVs secreted by cells growing in hypoxic conditions had a much higher level of the active form of the enzyme. The presence of a greatly elevated level of HIF1‐alpha in EVs from hypoxic cells confirms the myeloma cells response to their growth in hypoxic conditions.

**Figure 2:**
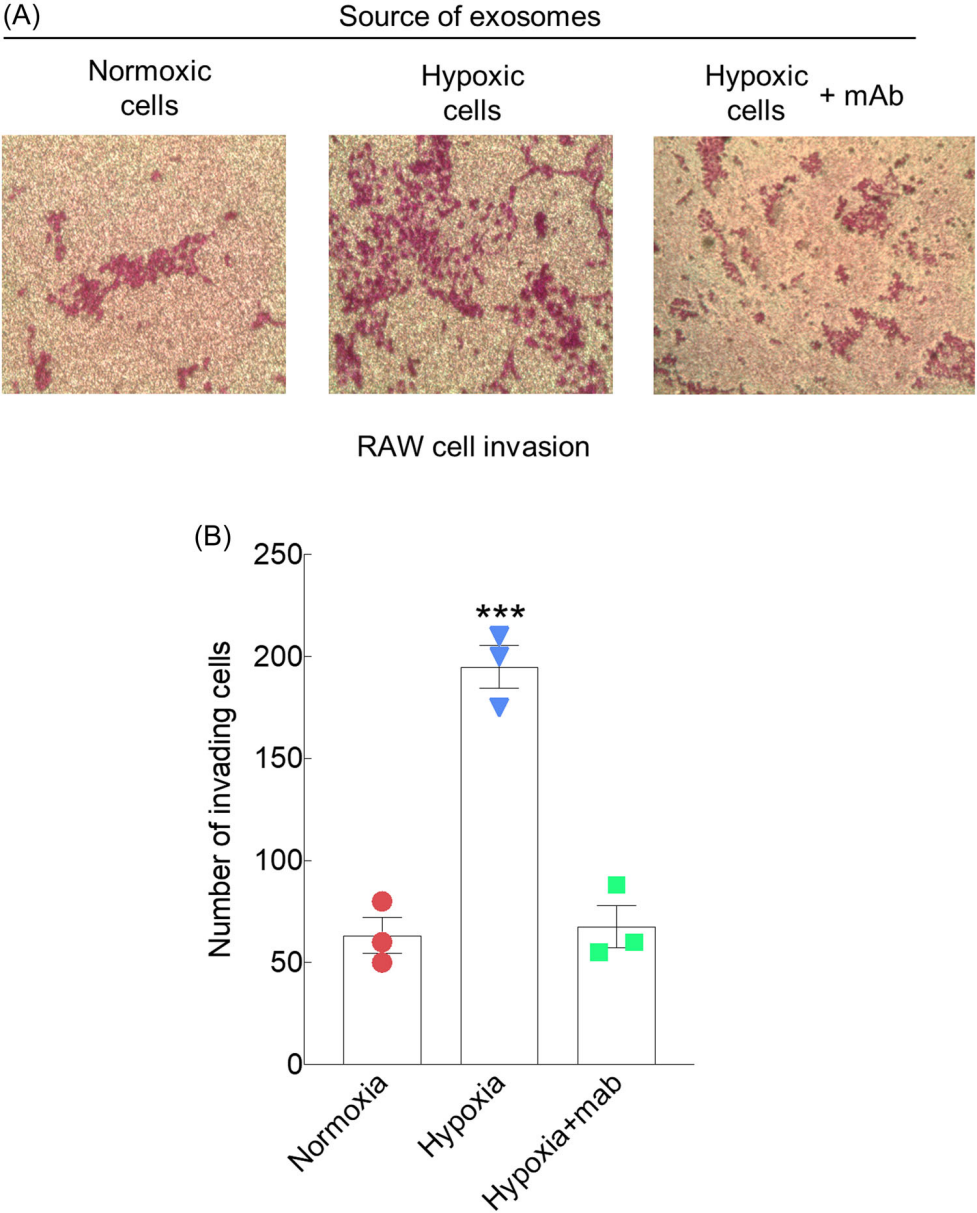
Inhibition of heparanase blocks the EV‐mediated increase in RAW cell invasion. (A) Invasion assays were performed as in Figure 1C in the presence or absence of H1023, a monoclonal antibody that blocks heparanase enzyme activity. (B) Three separate experiments were performed and the numbers of invading cells shown are from triplicate wells of a single representative experiment. ***p ≤ 0.001. EV, extracellular vesicle.

To determine if EVs released by hypoxic myeloma cells could influence cells within the tumor microenvironment, scratch assays were performed using RAW macrophages. Compared to EVs from cells growing in normoxic conditions, the EVs secreted by hypoxic myeloma cells significantly increased the migration of the macrophages following 14 h of exposure to the EVs (Figure 1B). Cells exposed to EVs from hypoxic cells had aggressively migrated into the scratch and almost completely covered the scratched area. Next, using a matrigel transwell assay, we tested whether EVs from hypoxic cells would enhance RAW cell invasion. Results demonstrated an almost fourfold increase in the number of invasive cells following exposure of cells to EVs from hypoxic cells compared to cells exposed to EVs from normoxic cells (Figure 1C). The role of HPSE in promoting the migration and invasion of endothelial cells, myeloma cells, and many other cancer cell types has been well documented.^12^, ^13^, ^28^, ^29^ We previously demonstrated that HPSE cargo in EVs can be transferred to recipient cells and impact their behavior.^14^, ^16^ Thus, we anticipated that HPSE in the EVs from cells growing in hypoxic conditions was mediating the enhanced macrophage invasion. To examine this, we exposed the RAW macrophages to EVs from hypoxic cells in the presence or absence of the HPSE‐inhibiting monoclonal antibody H1023.^30^, ^31^ In the presence of the antibody, invasion of the macrophages was dramatically reduced (Figure 2), confirming that EV HPSE was critical for driving the cells invasive behavior.

### EVs secreted by hypoxic cells enhance endothelial tube formation

**Figure 3:**
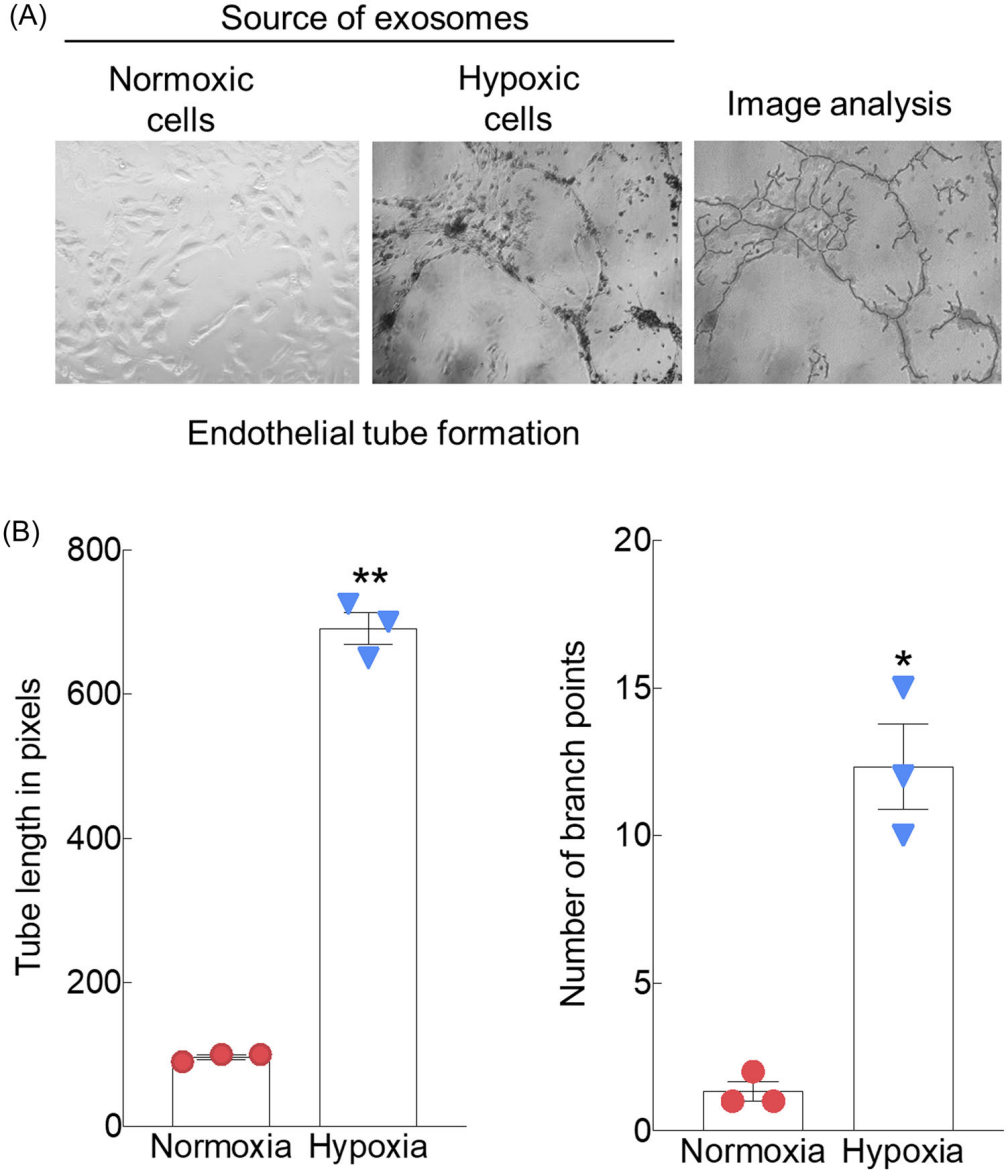
EVs secreted by hypoxic myeloma cells promote endothelial tube formation. (A) Endothelial tube formation assays were performed on matrigel coated wells utilizing human umbilical vein endothelial cells growing in the presence of EVs secreted by CAG‐HPSE cells grown in either normoxic or hypoxic conditions. Image analysis (right panel) by ImageJ software was utilized to determine (B) total length of tubes and number of branch points. **p  ≤  0.01 for tube length; *p  ≤  0.05 for number of branch points. Duplicate experiments were performed and shown are representative data from triplicate wells of a single experiment. EV, extracellular vesicle.

There is abundant evidence that HPSE aggressively promotes angiogenesis.^12^, ^13^ Our lab has studied this extensively in myeloma and demonstrated a pivotal role for HPSE in stimulating angiogenesis both in vitro and in vivo.^29^, ^32^, ^33^, ^34^ Thus, we speculated that the EVs generated in hypoxic conditions and having abundant HPSE cargo would stimulate endothelial tube formation. In the presence of EVs from normoxic cells, endothelial cells remained isolated as single cells and lacked tube formation entirely (Figure 3A,B). In contrast, in the presence of EVs from hypoxic cells, the endothelial cells aligned into tube‐like structures forming multiple branch points. Hypoxic EVs caused a dramatic increase in total tube length and number of branch points (nodes).

### EVs secreted by hypoxic cells promote spheroid formation by myeloma cells

**Figure 4:**
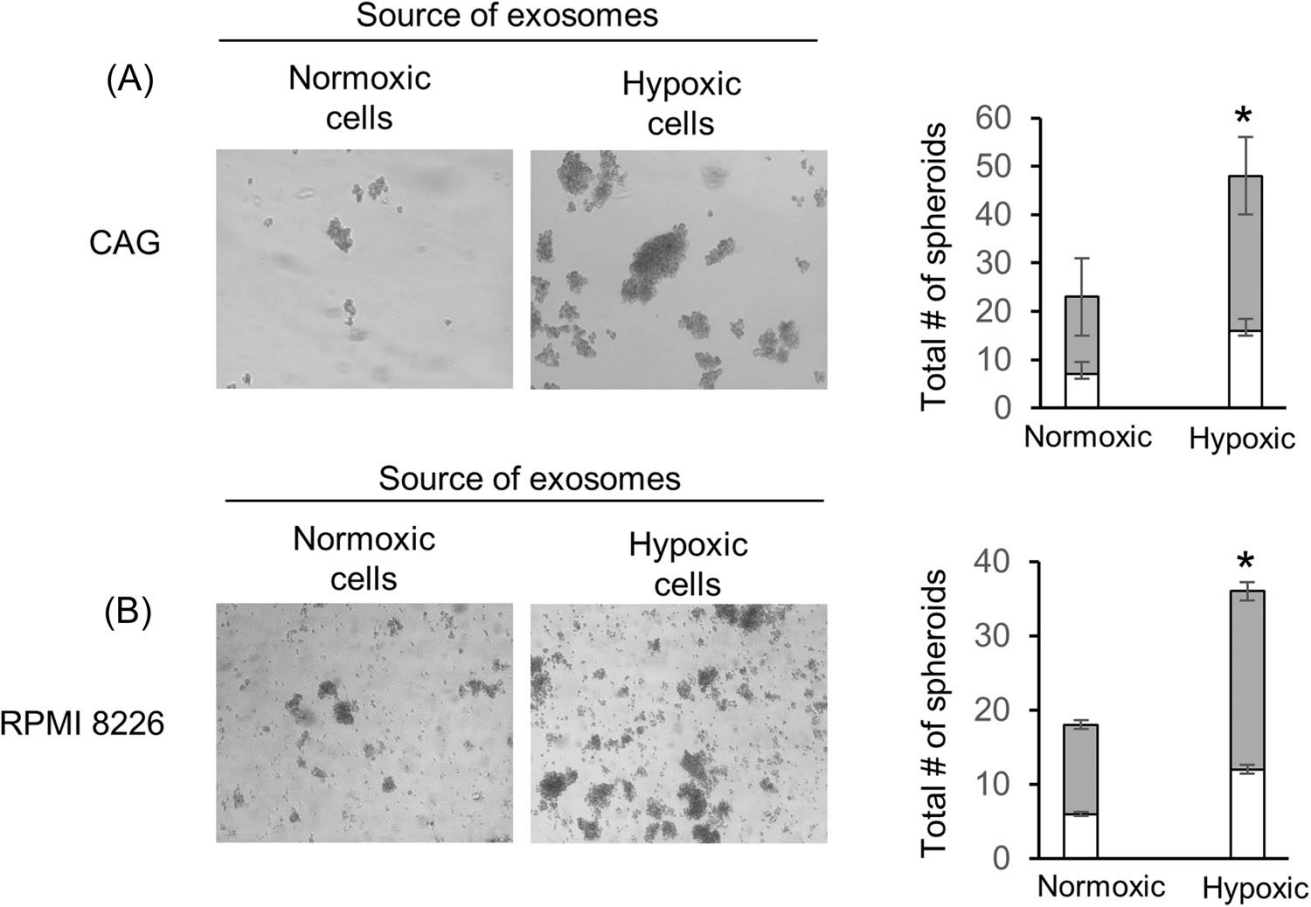
EVs secreted by hypoxic myeloma cells promote stemness. EVs secreted by CAG‐HPSE myeloma cells grown in either normoxic or hypoxic conditions were incubated for 7–10 days with (A) CAG (wild‐type) or (B) RPMI‐8226 human myeloma cell lines and their effect on spheroid formation assessed (representative photos shown, magnification, ×100). Bar graphs show quantification of spheres from three independent experiments is shown as mean ± SD. *p ≤ 0.05. White portion of bars in the graph represent the number of spheroids having less than 50 cells, gray portion of bars in the graph represent the number of spheroids having more than 50 cells. EV, extracellular vesicle.

We previously demonstrated that HPSE is capable of promoting myeloma cell stemness.^15^ When CAG‐HPSE myeloma cells were plated at low density and grown in serum‐free conditions that support survival and expansion of stem‐like cells, they formed more and larger tumor spheroids than did CAG cells expressing a low level of HPSE.^15^ Moreover, the addition of exogenous HPSE stimulated spheroid formation in CAG cells that expressed a low level of HPSE. Thus, we speculated that the EVs from cells grown in hypoxic conditions and having a high level of HPSE cargo (Figure 1A) would promote the stemness of CAG cells. To test this, EVs isolated from CAG‐HPSE cells growing in normoxic or hypoxic conditions were incubated with either wild‐type CAG or RPMI 8226 myeloma cells growing at low density and in serum‐free conditions. Results demonstrate that in both myeloma cell lines, spheroid formation dramatically increased in the presence of EVs from the hypoxic cells compared to cells growing in the presence of EVs secreted by normoxic cells (Figure 4).

## DISCUSSION

Using human myeloma cell lines, this work demonstrated a profound effect of hypoxia on the functional capacity of EVs secreted by these tumor cells. When compared to EVs secreted by cells growing in normoxic conditions, the EVs from hypoxic cells dramatically increased macrophage migration and invasion, enhanced endothelial tube formation and stimulated increased stemness properties of myeloma cells. Overall, this indicates that the hypoxic state can have broad impact due to its effect on the EVs secreted.

Although several studies have examined the impact of macrophage‐derived EVs on tumor cells, less is known about the effect of tumor EVs on macrophages.^35^, ^36^ Recent studies have demonstrated the ability of tumor‐derived EVs to regulate the phenotypic fate of macrophages by driving them to either the M1 or M2 state.^37^ This could be important to macrophage migration, as recent studies have shown that M2 macrophages have a greater capacity to migrate compared to M1 macrophages and this is related to integrin expression.^38^ In a separate study, it was revealed that colorectal cancer cells secrete EVs having miR‐934 as cargo that can be transferred to macrophages, induce the M2 phenotype and enhance cancer cell invasion.^39^ In the present study, we showed that hypoxia stimulates secretion of EVs that promote both migration and invasion of macrophages (Figures 1 and 2). It is worth noting that in this model EVs secreted by human myeloma cells interact with murine macrophages. EVs often display MHC/HLA molecules that could be involved in EV interaction with target cells.^40^ Thus, the incompatibility of these molecules in the human EV/mouse cell interaction used in our studies may impact activity of the macrophages.

Importantly, the EVs secreted by the myeloma cells have high levels of HPSE as cargo and blocking HPSE enzyme activity with antibody H1023 inhibits this EV‐mediated invasion by macrophages. The finding that H1023 blocks EV‐mediated invasion confirms the importance of HPSE in the invasive process of these cells. This HPSE activity may be the result of HPSE‐bearing exosomes transferring HPSE to the RAW cells or even due to exosome stimulation of HPSE expression by the RAW cells. Heparanase plays a key role in the invasion process by degrading heparan sulfate that is abundant in the extracellular matrix.^12^, ^41^ Also, once delivered to cells, HPSE can enhance expression of molecules that drive aggressive tumor behavior including vascular endothelial growth factor (VEGF) and hepatocyte growth factor, the latter being a stimulator of cell spreading.^42^ Importantly, HPSE can also stimulate shedding of the syndecan‐1 heparan sulfate proteoglycan and once shed, the syndecan‐1 can activate the Rac pathway to promote cell migration and invasion.^29^

In a previous study, we found that antimyeloma drugs when incubated with CAG myeloma cells stimulated a burst of exosome secretion and these exosomes had high levels of HPSE as cargo.^16^ We call these exosomes “chemoexosomes” to denote that they differ from exosomes secreted by cells not exposed to drug. Interestingly, in parallel to what we see with EVs from hypoxic cells, the chemoexosomes also stimulated macrophage invasion and this too could be inhibited by H1023 antibody. Thus, the myeloma cells responded similarly to both chemotherapy and hypoxia by secreting EVs high in HPSE that impact macrophage behavior. This further supports the use of anti‐HPSE drugs as anticancer agents. This may be particularly important in myeloma patients where hypoxic bone marrow niches often exist in the multiple lesions that are characteristic of this cancer.^43^ Roneparstat, a potent inhibitor of HPSE enzyme activity, has been shown to have antimyeloma activity in mouse models and was successfully tested in a phase I trial in myeloma patients.^34^, ^44^, ^45^ Moreover, roneparstat was effective in diminishing chemoresistance in a mouse model, suggesting that this drug may also aid in overcoming some of the negative effects of hypoxia.^46^

Numerous studies have revealed an upregulation of HPSE expression in cells growing in hypoxic conditions and some have linked that upregulation to an increase in cell invasion.^47^, ^48^, ^49^ There is also evidence linking hypoxia, HPSE, and upregulation of VEGF and increased angiogenesis. This is consistent with our finding that hypoxic EVs from myeloma cells promote the formation of endothelial tubes (Figure 3). In previous work, we demonstrated that upregulation of HPSE in the CAG cells enhances the amount of both HPSE and VEGF in the exosomes they secrete.^14^ Concentration of these factors in exosomes coupled with increased exosome secretion that occurs in hypoxic conditions^9^, ^10^ could dramatically drive angiogenesis within hypoxic tumor microenvironments.

Hypoxia often occurs in myeloma tumor lesions where tumor cells are growing in high density and blood flow is restricted.^50^ It is likely that these hypoxic niches harbor stem‐like cells that remain dormant and contribute to chemoresistance.^51^ It has been demonstrated that the growth of myeloma cell lines in hypoxic conditions promotes a stem‐like phenotype and rapidly renders cells resistant to commonly used antimyeloma drugs.^52^ We discovered here that EVs secreted by hypoxic myeloma cells could promote stemness properties in myeloma cells that were not growing in hypoxic conditions (Figure 4). This is important because it demonstrates that EVs secreted within a hypoxic niche could circulate within a patient and transfer information to recipient tumor cells located distally that confers upon those cells stem‐like properties. We have previously demonstrated that high levels of HPSE enhance myeloma stemness.^15^ In that study, the stemness phenotype was demonstrated by growing cells in stem cell‐forming conditions resulting in formation of spheroids. Moreover, the spheroids that formed from cells expressing high HPSE had elevated levels of glioma‐associated oncogene homologue 1, SRY‐box transcription factor 2, and aldehyde dehydrogenase 1 family, member A1, three genes known to be associated with myeloma stemness. Thus, it is likely that the EVs from hypoxic cells carrying a high level of HPSE cargo are, at least in part, promoting stemness by transferring HPSE to the recipient myeloma cells.

## CONCLUSIONS

The data presented here support the conclusion that when human myeloma cells are exposed to hypoxic conditions they secrete EVs that can dramatically help shape events within the tumor microenvironment. These EVs from hypoxic tumor cells carry an abundant level of the enzyme HPSE that is delivered to host cells within the microenvironment and influences their behavior, including promoting the migration of macrophages. Moreover, the EVs from hypoxic cells stimulated endothelial tube formation, indicating a potent ability to influence angiogenesis within tumor lesions. Importantly, because these effects are transmitted by EVs, it raises the possibility that they may impact tumor niches beyond the hypoxic environment from which they derive. Lastly, the observation that EVs from hypoxic cells can enhance tumor cell stemness contributes to our understanding of why myeloma tumors in patients remain resistant to therapy, eventually resulting in tumor relapse.

## AUTHOR CONTRIBUTIONS

Kaushlendra Tripathi conceived the work, designed, executed and analyzed the experiments. Shyam K Bandari contributed to the design and execution of the work, Ralph D Sanderson contributed to the design and analysis of experimental data. All authors contributed to the writing of the manuscript and approved the submitted version.

## CONFLICT OF INTEREST STATEMENT

The authors declare no conflict of interest.

## ETHICS STATEMENT

Not applicable.

## Data Availability

Data is available on request from the authors.
